# Supplementary material for: Ultra-high dynamic range quantum measurement retaining its sensitivity
Source: Nat Commun. 2021 Jan 12;12:306. doi: 10.1038/s41467-020-20561-x (PMC7804307; doi:10.1038/s41467-020-20561-x)
Supplement: Supplementary file 1 — Supplementary Information [file 41467_2020_20561_MOESM1_ESM.pdf]

Ultra-high dynamic range quantum measurement retaining its sensitivity

E. D. Herbschleb[1], H. Kato[2], T. Makino[2], S. Yamasaki[2], N. Mizuochi[1]
[1]*Institute for Chemical Research, Kyoto University, Gokasho, Uji-city, Kyoto 611-0011, Japan*
[2]*National Institute of Advanced Industrial Science and Technology (AIST), Tsukuba, Ibaraki 305-8568, Japan*

Contents

Supplementary Note 1: Maximum uncertainty 2
Supplementary Note 2: Homogeneity of uncertainty σ_B 3
Supplementary Note 3: On scaling (of uncertainty with respect to measurement time) and limits 6
Supplementary Note 4: Effect of additional areas 8
Supplementary Note 5: Measurement details 9
Supplementary Note 6: Extension of the range beyond the standard measurement's range 10
Supplementary Note 7: Algorithm implementation 12
Supplementary Note 8: Small area measurements for AC 13
Supplementary Note 9: Single result vs probability distribution 16

Supplementary Note 1: Maximum uncertainty

Given a Hahn-echo sequence, the period in magnetic field amplitude $B_{\text{AC, period}}$ is (see Supplementary Note 5 of [1])

$$B_{\text{AC, period}} = \frac{2\pi h}{4 g \mu_{\text{B}} t}, \tag{1}$$

with h Planck's constant, g the g-factor of the electron spin, μ_{B} the Bohr magneton and t the time delay between the $\pi/2$-pulses. Here, the range B_{range} of a measurement is half this period (illustrated in Supplementary Fig. 1a), which can be extended to the whole period by combining it with a measurement which has its result shifted by a non-zero phase (ideally $\pi/2$, see for example Supplementary Fig. 7a).

Given a probability distribution $P(B)$ within B_{range} for the magnetic field B, the standard deviation is

$$\sigma_{\text{B}} = \sqrt{\int_0^{B_{\text{range}}} P(B) \left(B - B_{\text{expected}}\right)^2 dB}, \tag{2}$$

with B_{expected} the expected value

$$B_{\text{expected}} = \int_0^{B_{\text{range}}} P(B)\, B dB. \tag{3}$$

This standard deviation is the uncertainty in the magnetic field. Please note that the distributions for these equations have to be normalised, but they are displayed throughout the paper and supplementary without normalisation. Also, since the field is determined only within a range due to periodicity, the expected value and standard deviation are calculated after centring the distribution around its maximum (around the expected value would be better, but the maximum and expected value are nearly the same generally).

Suppose we have no knowledge about the applied field, then the probability distribution is flat within its whole range, thus $P(B) = 1/B_{\text{range}}$ and $B_{\text{expected}} = B_{\text{range}}/2$. This gives for the uncertainty

$$\sigma_{\text{B}} = \sqrt{\frac{1}{B_{\text{range}}} \int_0^{B_{\text{range}}} \left(B - \frac{B_{\text{range}}}{2}\right)^2 dB} = \sqrt{\frac{1}{B_{\text{range}}} \left[\frac{1}{3}\left(B - \frac{B_{\text{range}}}{2}\right)^3\right]_0^{B_{\text{range}}}} = \frac{B_{\text{range}}}{\sqrt{12}} = \sigma_{B,\,\text{max}}. \tag{4}$$

This is the maximum possible uncertainty $\sigma_{B,\,\text{max}}$, as shown in Supplementary Fig. 1b.

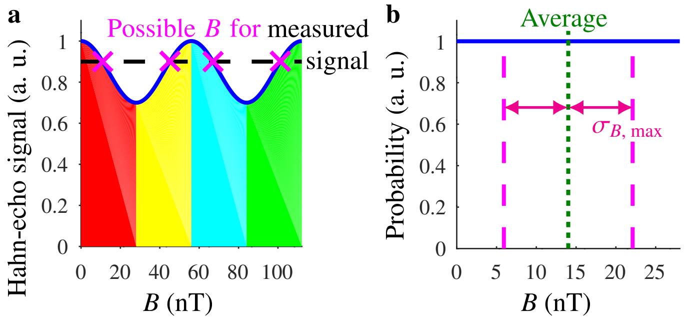

Supplementary Fig. 1. Maximum uncertainty. **a** The oscillating signal (blue line), which oscillates due to the rotation of the spin in the Bloch sphere given the magnetic field B, uniquely defines the result of a measurement to one of the ranges indicated with different colours only. An example measured signal (black dashed line) would have a solution (magenta cross) in every range. **b** When the field is not known at all, so the probability distribution (blue line) is flat within the chosen range, the uncertainty σ_B reaches its maximum (indicated with vertical magenta dashed lines). Here, the expected value for the field B_{expected} is simply the average.

Supplementary Note 2: Homogeneity of uncertainty σ_B

For a number of measurement sequences, the uncertainty σ_B (defined by Supplementary Equation (2)) is simulated in the range of the respective measurements, and the results are shown in Supplementary Fig. 2. For the standard measurement (red line in Supplementary Fig. 2), the uncertainty is not homogeneous across its range. Even at the most sensitive point, it is worse than the uncertainty usually reported (red cross in Supplementary Fig. 2a), since the latter results from linearising the sinusoid (for example the blue line in Supplementary Fig. 1a) at its maximum gradient. Please note that this sinusoid is the measured signal S for a Hahn-echo sequence. This inhomogeneity is easy to understand when approximating the uncertainty in S as a normal distribution (which is a fairly accurate approximation of the actual Poisson distribution with applied continuity correction when receiving more than ~ 10 photons, which is the case for relevant measurement times), while ignoring the difference in shot-noise between the read-out of the $|0\rangle$ and $|1\rangle$ states for now. This gives a (non-normalised) probability distribution

$$P(S) = \exp\left(-\frac{\left(S - S_{\text{exp}}\right)^2}{2\sigma_S^2}\right), \tag{5}$$

with S_{exp} the signal expected to measure given the actual magnetic field, and σ_S its standard deviation. The relation between the signal and the magnetic field B is (due to the rotating spin, see Supplementary Note 5 of [1])

$$S(B) = A\cos(\omega_B B + \phi) + O, \tag{6}$$

with A the amplitude, ω_B the frequency in magnetic field, ϕ a phase, and O an offset. Thus, the (non-normalised) distribution for the field is

$$P(B) = \exp\left(-\frac{(A\cos(\omega_B B + \phi) - A\cos(\omega_B B_{\text{act}} + \phi))^2}{2\sigma_S^2}\right) = \exp\left(-\frac{(\cos(\omega_B B + \phi) - \cos(\omega_B B_{\text{act}} + \phi))^2}{2(\sigma_S/A)^2}\right), \tag{7}$$

with B_{act} the actual magnetic field which thus gives the expected S_{exp}. It is clear that the shape of the resulting distribution depends on the actual magnetic field, and hence the uncertainty σ_B depends on the magnetic field as well.

Next, we combine two measurements which have their measured signals $\pi/2$ apart, here we choose $\phi = 0$ and $\phi = -\pi/2$. Distributing the measurement time equally amongst them (thus the uncertainty in the measured signal is $\sqrt{2}$ worse), this gives the combined (non-normalised) distribution

$$\begin{aligned}
P(B) &= \exp\left(-\frac{(\cos(\omega_B B) - \cos(\omega_B B_{\text{act}}))^2}{2\left(\sqrt{2}\sigma_S/A\right)^2}\right)\exp\left(-\frac{(\sin(\omega_B B) - \sin(\omega_B B_{\text{act}}))^2}{2\left(\sqrt{2}\sigma_S/A\right)^2}\right) \\
&= \exp\left(-\frac{\cos^2(\omega_B B) - 2\cos(\omega_B B)\cos(\omega_B B_{\text{act}}) + \cos^2(\omega_B B_{\text{act}}) + \sin^2(\omega_B B) - 2\sin(\omega_B B)\sin(\omega_B B_{\text{act}}) + \sin^2(\omega_B B_{\text{act}})}{2\left(\sqrt{2}\sigma_S/A\right)^2}\right) \\
&= \exp\left(-\frac{2 - 2\cos(\omega_B(B - B_{\text{act}}))}{2\left(\sqrt{2}\sigma_S/A\right)^2}\right).
\end{aligned} \tag{8}$$

The shape (around B_{act}) of this distribution, and hence the uncertainty, is independent of the actual magnetic field (blue line in Supplementary Fig. 2a and b). Please note that this constant uncertainty is lower than the maximum uncertainty of the standard single-phase measurement, meaning that it guarantees a lower uncertainty, and is thus superior for large-range measurements.

When taking the difference in uncertainty of the signal into account for the different states, hence creating an asymmetric change in uncertainty around the mean (the superposition state $1/\sqrt{2}(|0\rangle + |1\rangle)$), the uncertainty is not homogeneous any more (cyan line in Supplementary Fig. 2). However, this is improved by combining four measurements with their signals all $\pi/2$ apart (magenta line in Supplementary Fig. 2). If even more homogeneity is required, more phases can be added, each pair of phases $\pi/2$ apart (for example the black dashed line in Supplementary Fig. 2). The two-phase and four-phase versions are the basic ones: one pair and two pairs; each pair ensures to support the range of the whole period, and homogeneity for white noise.

When applying a Poisson distribution instead of the normal approximation, the results are very similar (dark blue dashed line for two phases, and green dashed line for four phases in Supplementary Fig. 2), justifying the approximation. Adding quantum-projection noise to the four-phase case (purple dashed line in Supplementary Fig. 2) illustrates

that it is negligible. Therefore, in all simulations throughout the paper and supplementary notes, the uncertainty σ_S in the measured signal is approximated with a normal distribution for shot-noise

$$\sigma_S = \frac{1}{\sqrt{N N_{\text{ph}}}}, \tag{9}$$

with N the number of iterations of the measurement sequence and N_{ph} the number of photons per iteration [1].

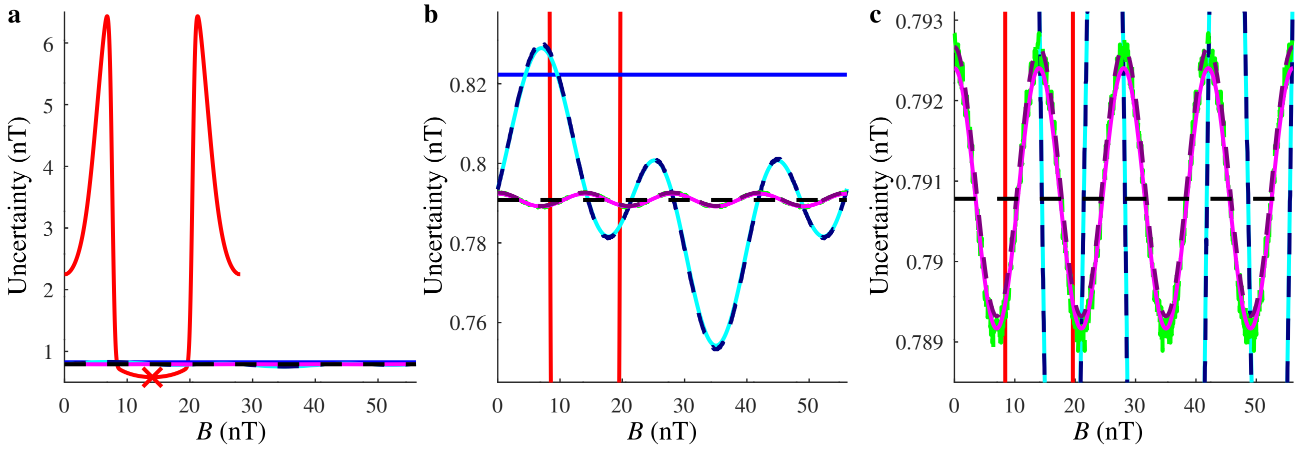

Supplementary Fig. 2. Uncertainty homogeneity. **a, b, c** Different zoom levels of simulations of uncertainty vs magnetic field amplitude B as explained in the text: single phase (red line), uncertainty given by standard formula (red cross), two phases (upper blue line), two phases with asymmetric noise (cyan line), two phases with Poisson distribution (dark blue dashed line on top of cyan line), four phases with asymmetric noise (magenta line), four phases with Poisson distribution (green dashed line underneath magenta line, mostly visible in **c**), four phases with added quantum-projection noise (purple dashed line slightly above magenta line), eight phases with asymmetric noise (black dashed line). The simulation without taking asymmetric noise into account slightly overestimates the uncertainty. Please note that the chosen simulated measurement time puts the uncertainties far below their maximum (see Supplementary Note 1), and the sensitivity is proportional to the uncertainty. Also, the standard uncertainty (red cross) is smaller than the actual one for the standard single-phase measurement (red line), since the former is computed by linearising the sinusoid at its maximum gradient. Only at infinite measurement time, the curve and cross coincide.

If there is quantum-projection noise only, actually, a single phase suffices. The probability to measure $|0\rangle$ is

$$p_{|0\rangle} = \cos^2\left(\frac{\theta}{2}\right), \tag{10}$$

with θ the angle between the z-axis and the spin in the Bloch sphere. For the measurement giving signal S from Supplementary Equation (6), this is

$$\theta = \text{acos}\left(\frac{S-O}{A}\right) = \omega_B B + \phi. \tag{11}$$

The uncertainty in field is estimated via the gradient grad in a point given the uncertainty in the signal (see Supplementary Note 5 of [1])

$$\sigma_B = \frac{\sigma_S}{|\text{grad}|}, \tag{12}$$

where grad follows from the derivative of the signal

$$\text{grad} = \frac{\partial S}{\partial B} = -\omega_B A \sin(\omega_B B + \phi). \tag{13}$$

Thus, the uncertainty for quantum-projection noise is (for N iterations)

$$\begin{aligned}\sigma_B = \frac{\sigma_S}{|\text{grad}|} &= \frac{\frac{\sqrt{p_{|0\rangle}(1-p_{|0\rangle})}}{\sqrt{N}}}{|\omega_B A \sin(\omega_B B + \phi)|} = \frac{\sqrt{\cos^2\left(\frac{\theta}{2}\right)\left(1-\cos^2\left(\frac{\theta}{2}\right)\right)}}{\sqrt{N}\omega_B A\,|\sin(\omega_B B + \phi)|} = \frac{\sqrt{\cos^2\left(\frac{\theta}{2}\right)\sin^2\left(\frac{\theta}{2}\right)}}{\sqrt{N}\omega_B A\,|\sin(\omega_B B + \phi)|} \\ &= \frac{\sqrt{\sin^2(\theta)}}{\sqrt{4N}\omega_B A\,|\sin(\omega_B B + \phi)|} = \frac{|\sin(\theta)|}{\sqrt{4N}\omega_B A\,|\sin(\omega_B B + \phi)|} = \frac{|\sin(\omega_B B + \phi)|}{\sqrt{4N}\omega_B A\,|\sin(\omega_B B + \phi)|}.\end{aligned} \tag{14}$$

Since the sinusoids cancel, this is independent of the field. Thus, adaptive measurements would not improve the uncertainty in this case.

As a final remark, we look at the Taylor expansion of the exponent in Supplementary Equation (8). This gives

$$2 - 2\cos(\omega_B\,(B - B_{\mathrm{act}})) = 2\sum_{n=1}^{\infty} \frac{(-1)^{n-1}\,\omega_B^{2n}}{(2n)!}\,(B - B_{\mathrm{act}})^{2n}\,. \tag{15}$$

For just $n = 1$, this gives the third order Taylor expansion (zeroth and all odd orders are zero), in which case the distribution is

$$P\,(B) \approx \exp\left(-\frac{\omega_B^2\,(B - B_{\mathrm{act}})^2}{2\left(\sqrt{2}\sigma_S\,/A\right)^2}\right) = \exp\left(-\frac{(B - B_{\mathrm{act}})^2}{2\left(\sqrt{2}\sigma_S\,/\,(A\omega_B)\right)^2}\right). \tag{16}$$

Therefore, the result is approximately normal with an uncertainty of the measured signal $\sqrt{2}$ worse compared to the linearised standard uncertainty (the latter being $\sigma_B = \sigma_S\,/\mathrm{grad}_{\mathrm{max}}$ with the maximum gradient of the sinusoid $\mathrm{grad}_{\mathrm{max}} = A\omega_B$, the red cross in Supplementary Fig. 2a). This is visible in Supplementary Fig. 2a by comparing the blue line with the red cross, however, the former's uncertainty is homogeneous, lower than the maximum standard uncertainty, and its range is twice as large. Moreover, the dynamic range, which is the important ratio between the maximum range and the sensitivity, is better for the multi-phase measurement than for the standard measurement by $\sqrt{2}$ (since the former has double the range), even when the latter's minimum uncertainty would be reached by for example adaptive measurements. Potentially, choosing two phases with a small effective offset (so close to $n\pi$ with n any integer, but not equal to it) might improve it for long measurement times (since then the range doubles with an uncertainty better than non-adaptive, but by less than a factor of $\sqrt{2}$), but worsens it for short measurement times (given the broadened peak created by two neighbouring sub-peaks). We did not investigate this new advanced type of adaptive measurement, thus whether it would be an improvement at reasonable measurement times and how much additional overhead is required (for example to determine the offset in case it should be updated as well during an optimal measurement) remain unknown.

For further study of the homogeneity of uncertainties, we recommend [2], which gives an excellent theoretical description of previous algorithms, including their homogeneity. Please note that although details differ, the important point that the homogeneity of the uncertainty improves with increasing number of phases, is the same.

Supplementary Note 3: On scaling (of uncertainty with respect to measurement time) and limits

Below we give our view on scaling of the uncertainty with respect to the measurement time T_{meas}, and the lower limits of the uncertainty using a single NV centre. It is important to realise that the absolute values of these uncertainties depend on the method of measuring the spin, here by a standard measurement (a single laser pulse). Changing this method alters these absolute values (in theory, in simulation and in measurement), but not the relative values and thus conclusions. Moreover, only the time delay between the $\pi/2$-pulses in a sequence is taken into account as the measurement time to focus on the effect of the sensing part of the measurement.

In Supplementary Fig. 3, the uncertainty is plotted for different measurement sequences for AC sensing. Please note that the details depend on the frequency, but the general results remain similar; here the optimum frequency is chosen for $T_2 = 2.0$ ms (see Supplementary Information 5 of [1]). The text below describes the results of this figure.

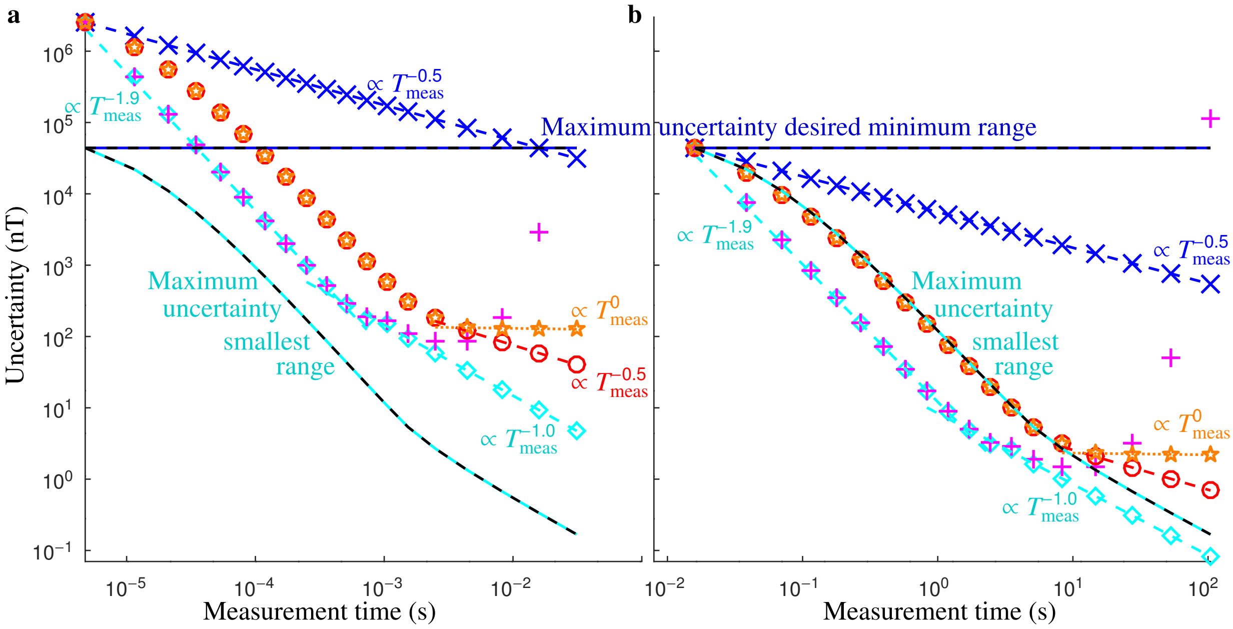

Supplementary Fig. 3. Uncertainty vs measurement time T_{meas} for AC sensing. Cyan diamonds: increasing the time delay between the $\pi/2$-pulses continuously, giving the so-called Heisenberg limit. This does decrease the range, thus only the leftmost diamond supports the desired range. T_2 is infinite. Magenta pluses: as cyan diamonds, but $T_2 = 2.0$ ms, thus the uncertainty increases rapidly for long measurement times. Blue crosses: repetition of the sequence that gives the desired minimum range. Orange pentagons: the (sub)sequence supporting the desired range remains part of the total sequence, while a subsequence for half the previous area is added every time. Therefore, the desired range remains supported in an efficient way. For long measurement times, the added longer delay subsequences do not lower the uncertainty significantly, hence it levels off. Red circles: as orange pentagons, but instead of adding subsequences with longer delays after the level-off point, only the subsequence with the optimum delay is added continuously. Horizontal black-blue line: maximum uncertainty given the desired range (see Supplementary Note 1). Diagonal black-cyan line: maximum uncertainty given the limited range of the sequence used for the cyan diamonds. **a** Each sequence is run a single time, unless at the repeating stage (which is always for blue crosses, and for long measurement times for red circles). Owing to the chosen measurement method, the measured uncertainty is usually above the maximum uncertainty. **b** Each sequence is repeated sufficiently to lower the measured uncertainty below the range-defined maximum uncertainty. This gives the limit for the chosen measurement method.

The cyan diamonds indicate what is often called the Heisenberg limit. By simply increasing the time delay between the $\pi/2$-pulses in the sequence, the uncertainty becomes smaller. Initially, it scales as $T_{\text{meas}}^{-1.9}$, while at the end it scales as T_{meas}^{-1}. This follows directly from integrating a sinusoid: for short times the sinusoid approaches a straight line (hence the quadratic relation, explained in main text Fig. 1c as well, thus eventually scaling as T_{meas}^{-2}), for long measurement times a periodic function (hence the linear relation).

There are some notes for this limit though: the coherence time T_2 is infinite, and the range of the measurement becomes smaller with decreasing uncertainty (as described in the main text). Taking the limited T_2 into account results in the magenta pluses, which uncertainty quickly grows for $T_{\text{meas}} > T_2$. To ascertain the required range, for longer measurement times, the simplest method is to repeat the measurement sequence with this range, resulting in the blue crosses which has scaling $T_{\text{meas}}^{-0.5}$. This is simply statistical averaging (central limit theorem).

To find the limit taking into account the desired range, the sequence with the largest range is performed once, then the sequence with half that range (but lower uncertainty) is added, and so on (as implied by the optimum in the main text). This results in the orange pentagons, which scale less steeply than the Heisenberg limit, since it keeps approaching it. For long measurement times it scales as T^0_{meas}, because the limited T_2 means that the added sequences at these times do not contribute to lowering the uncertainty. This is addressed by repeating the sequence with the optimal time delay instead of adding sequences with longer time delays, which results in the red circles.

However, Supplementary Note 1 derived the maximum uncertainty given the supported range, which is plotted as blackish lines. The horizontal one is the maximum uncertainty for the desired range, while the diagonal one is the maximum uncertainty for the range of sequences giving the Heisenberg limit (cyan diamonds). Apparently, not measuring at all gives a smaller uncertainty for the former at short measurement times, and for the latter always. To make the measurement functional, its uncertainty should be at least below this maximum uncertainty, thus each (sub)sequence needs to be measured more than once. Supplementary Fig. 3b shows the same results as in Supplementary Fig. 3a, except that for every measurement time, starting at the shortest one possible given the desired range, the number of times each sequence is repeated is such that the uncertainty after measuring is at maximum this maximum uncertainty (with equal repetitions for each subsequence). This would be the limiting uncertainty, with the red circles giving an approximate limit for large-range measurements: for long measurement times it is asymptotically correct, but for short measurement times the assumption of equal repetitions of the subsequences is not optimal, and hence the lowest uncertainty possible is probably lower (but higher than the small-range Heisenberg limit). Main text Fig. 2b shows this behaviour.

It is worth noting that the blue crosses exactly align in Supplementary Fig. 3a and b, since this was a repetition of the same sequence already. Also, the maximum uncertainty related to the desired range remains the same. More importantly, the red circles (the limiting uncertainty for large-range measurements) at long measurement times (so when it scales as $T^{-0.5}_{\text{meas}}$) are also nearly a continuation from Supplementary Fig. 3a to b. This is since only the last subsequence with the optimal time delay is repeated in the end, hence the overhead of the subsequences with shorter delays becomes negligible at long measurement times.

This reveals an important point for coherence-limited measurements: for long measurement times the lowest possible uncertainty is limited by the coherence time and thus follows directly from this. Hence, for long measurement times, the limit scales as $T^{-0.5}_{\text{meas}}$ along the repetition of the sequence with the optimal delay. When moving to shorter measurement times, eventually the scaling should become Heisenberg-like. However, as long as the uncertainty is not on top of this limit, the steeper the scaling is, the slower it approaches this limit. Moreover, when an algorithm reaches the Heisenberg-like scaling before reaching the limit, it will never reach the limit. Thus, for measurement algorithms, generally the steeper the dependency, the worse the algorithm. Fig. 3a in the main text displays some clear examples.

Finally, for completeness, Supplementary Fig. 4 gives the results for DC sensing. The main differences are that the Heisenberg limit is less steep, it follows a single line (T^{-1}_{meas}), and the small-range and large-range limits are closer together. These follow from the linear time-delay vs area relation (see main text Fig. 1c).

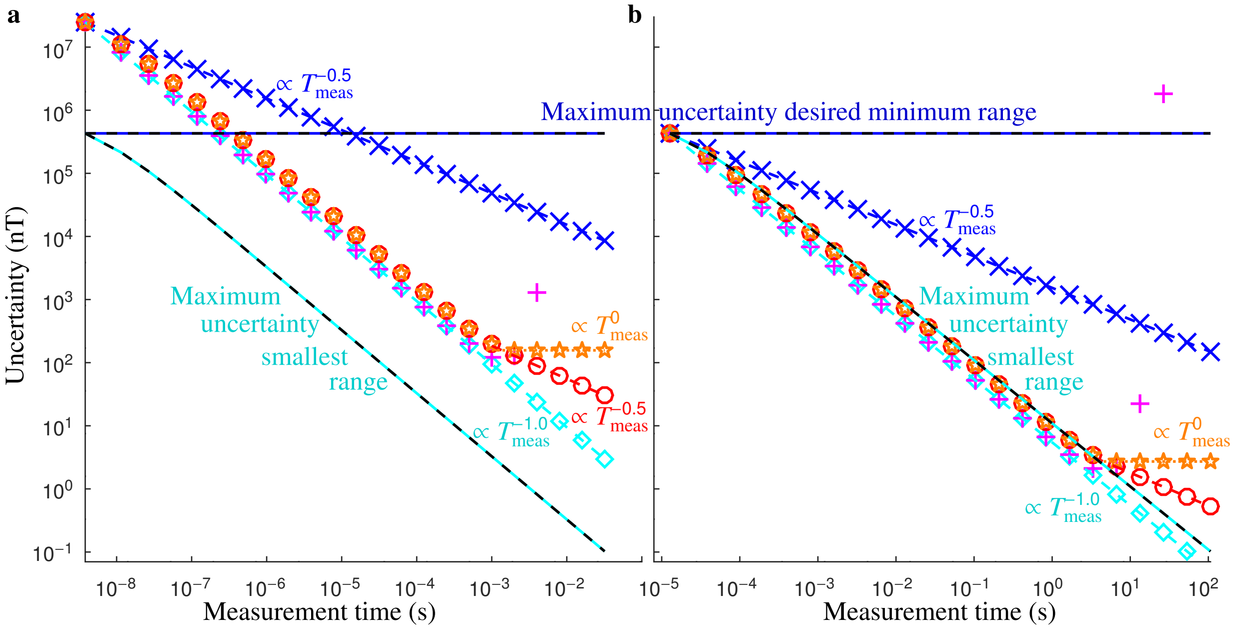

Supplementary Fig. 4. Uncertainty vs measurement time for DC sensing. **a/b** Analogous to Supplementary Fig. 3.

Supplementary Note 4: Effect of additional areas

To get an idea of the effect on the uncertainty of added areas in a sequence, we simulate a three-area sequence with halved areas: A_0, $A_0/2$ and $A_0/4$. Then, we add one more area at any integer multiple of the smallest area in order to keep the range the same (thus at $nA_0/4$ with $n = 1, 2, 3, 4$; Supplementary Note 6 explains more about the range), and we find the optimum relative number of iterations for all four cases. To look at this effect by itself, this is simulated without taking overhead time into account (see Supplementary Note 8). The result is shown in Supplementary Fig. 5a. It indicates that probably, the more areas the lower the uncertainty. Additionally, a similar simulation is performed for a four-area sequence (A_0, $A_0/2$, $A_0/4$ and $A_0/8$), thus by adding $nA_0/8$ with $n = 1 - 8$, which shows a similar result (Supplementary Fig. 5b).

To further clarify the effect, Supplementary Fig. 5c plots the uncertainty-time relation for sequences with the same range, but with increasingly more areas added. The steep region is mostly effected by these added areas, while the rest is fairly similar.

Thus the effect of these extra areas is small, but if utilised, please note that for AC, adding areas between half the period and the full period of the sinusoid is only feasible if the rest of the period can be used for smaller areas.

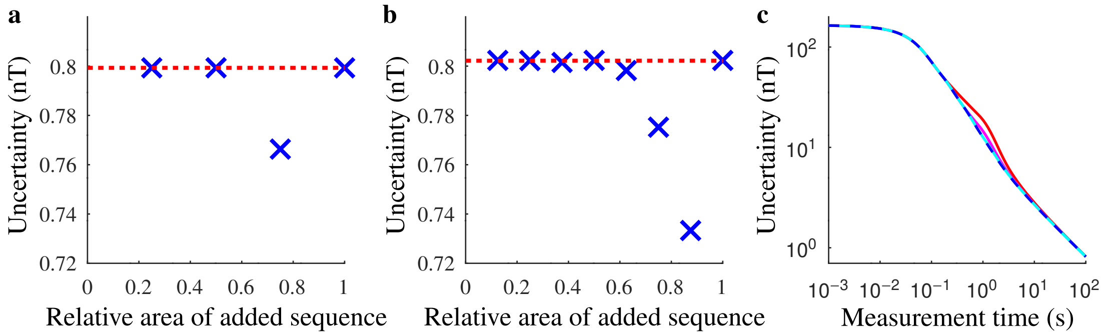

Supplementary Fig. 5. Optimum amount of areas. **a** Uncertainty vs relative area of the added sequence. The subsequences for areas $nA_0/4$ with $n = 1, 2, 4$ are always part of the total sequence, while a subsequence for area $nA_0/4$ for $n = 1, 2, 3, 4$ is added (thus potentially adding the same subsequence), with $n/4$ given on the horizontal axis and the uncertainty for each given by blue crosses. The optimum uncertainty without added sequence is indicated with a red dotted line as guide to the eye. As expected, the uncertainty for having a sequence twice remains the same for the optimum relative number of iterations, while for $3A_0/4$ the uncertainty slightly decreases, indicating that the more different areas are added, the lower the uncertainty in the optimum. **b** As **a** but for areas $nA_0/8$ with $n = 1, 2, 4, 8$ always part of the sequence, and area $nA_0/8$ for $n = 1 - 8$ added. **c** Uncertainty vs measurement time for two areas (red line with highest uncertainty: A_0 and $A_0/8$), three areas (magenta line between the red and blue lines: A_0, $A_0/4$ and $A_0/8$), four areas (cyan line under the blue dashed line: A_0, $A_0/2$, $A_0/4$ and $A_0/8$) and five areas (blue dashed line: A_0, $3A_0/4$, $A_0/2$, $A_0/4$ and $A_0/8$). Adding more areas mostly effects the steep area; the difference between the cyan line and blue dashed line is rather small.

Supplementary Note 5: Measurement details

For the measurement data of Fig. 2b in the main text, the relative number of iterations is optimised for each measurement time. For completeness, these are given in Supplementary Fig. 6. The shortest measurement time is such that the steep region has just been entered (thus two areas contribute), and the longest is chosen to be just after exiting the steep region (thus the largest area becomes dominant). The choice was made to use quartered areas to limit the simulation time (which, without optimisations, increases exponentially with the number of areas). This choice slightly effects the uncertainty only, as visible in Supplementary Fig. 5c. Please note that the relative number of iterations for each area depends on the quantum system and the choice of areas (thus maximum range), and should be calculated for each case. Of course, given a specific sensor, these need to be calculated once only.

For optimising the relative number of iterations, we use a simple brute-force searching algorithm. Given the shape of the curves of the relative number of iterations with respect to measurement time (monotonically increasing), there is significant room to improve the optimising algorithm.

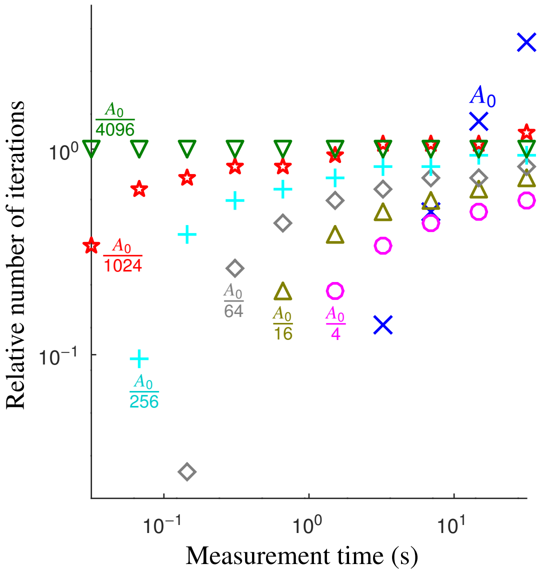

Supplementary Fig. 6. Measurement details. Relative number of iterations for each area in the sequence for the measurement of main text Fig. 2b: largest area A_0 (blue crosses), area $A_0/4$ (magenta circles), area $A_0/16$ (olive up-pointing triangles), area $A_0/64$ (grey diamonds), area $A_0/256$ (cyan pluses), area $A_0/1024$ (red pentagons), and area $A_0/4096$ (green down-pointing triangles). The latter are kept at 10^0.

Supplementary Note 6: Extension of the range beyond the standard measurement's range

It is possible to extend the range beyond what is attainable with a standard measurement that has the shortest possible time delay between the $\pi/2$-pulses, by combining areas that are not integer multiples of each other. Since each area is proportional to the frequency in signal with respect to the magnetic field (ω_B in Supplementary Equation (6)), the period (and hence range) of the combination of areas follows from the greatest common divisor (not necessarily integers) of the frequencies of all applied areas, as illustrated in Supplementary Fig. 7a.

In principle, the extension of the range is unlimited if any time delay can be set (even if there is a minimum time delay). However, since in experiments these step sizes are set digitally, they are restricted to multiples of the time resolution. Therefore, in such a case, the largest extension of the range follows from the time resolution which thus measures the smallest area, since all other areas are multiples of this area. Even if the time delay between the pulses is required to be longer, for example due to switching delays (to change the phase of the microwave), the range can still be extended to this maximum by choosing areas with frequencies that have this minimum frequency as greatest common divisor. This is always possible with prime factors: for example if 1 Hz is not attainable, combining 13 Hz and 17 Hz still gives 1 Hz as the overall frequency.

Moreover, by changing the frequency in different ways, for example with timed pulses, any range is possible. The implementation is sensor and application dependent. Apart from precisely timed pulses, any sort of signal scaling would work for any system. Specifically for AC, it is possible to move to a different location on the sinusoid curve, since this would change the area in a non-linear way (and thus the frequency as well).

The effect on the uncertainty is displayed in Supplementary Fig. 7b. It is still possible to get arbitrarily close to the ultimate uncertainty, but slightly longer measurement times are required compared to using smaller areas. The difference in relative differences is about a factor of 2 for a given measurement time, and keeps decreasing for longer measurement times (which makes sense, since the largest area keeps increasing in relative importance). From different simulations, it seems that this approximate factor of 2 might be the square root of the factor between the smallest areas of each sequence (so $\sqrt{(A_0/5)\,/\,(A_0/20)}$), but a more in-depth analysis is required to find a proper description. Please note that this is in relative difference with the ultimate uncertainty, in absolute value the uncertainties are rather similar (at long measurement times). Also, since the smallest areas complement each other to increase the range, they do not turn on after each other, such as in Fig. 3c in the main text, but essentially act as a combined area. For completeness, the reason for the perhaps unexpected domination of the larger area (of the two smallest areas) over the smaller area at short measurement times (as opposed to Fig. 3c in the main text, where the smallest area dominates) is simply since a distribution with an even number of evenly distributed peaks has always a larger uncertainty than a distribution with an odd number of evenly distributed peaks (which approaches the even uncertainty from below with an increasing number of peaks).

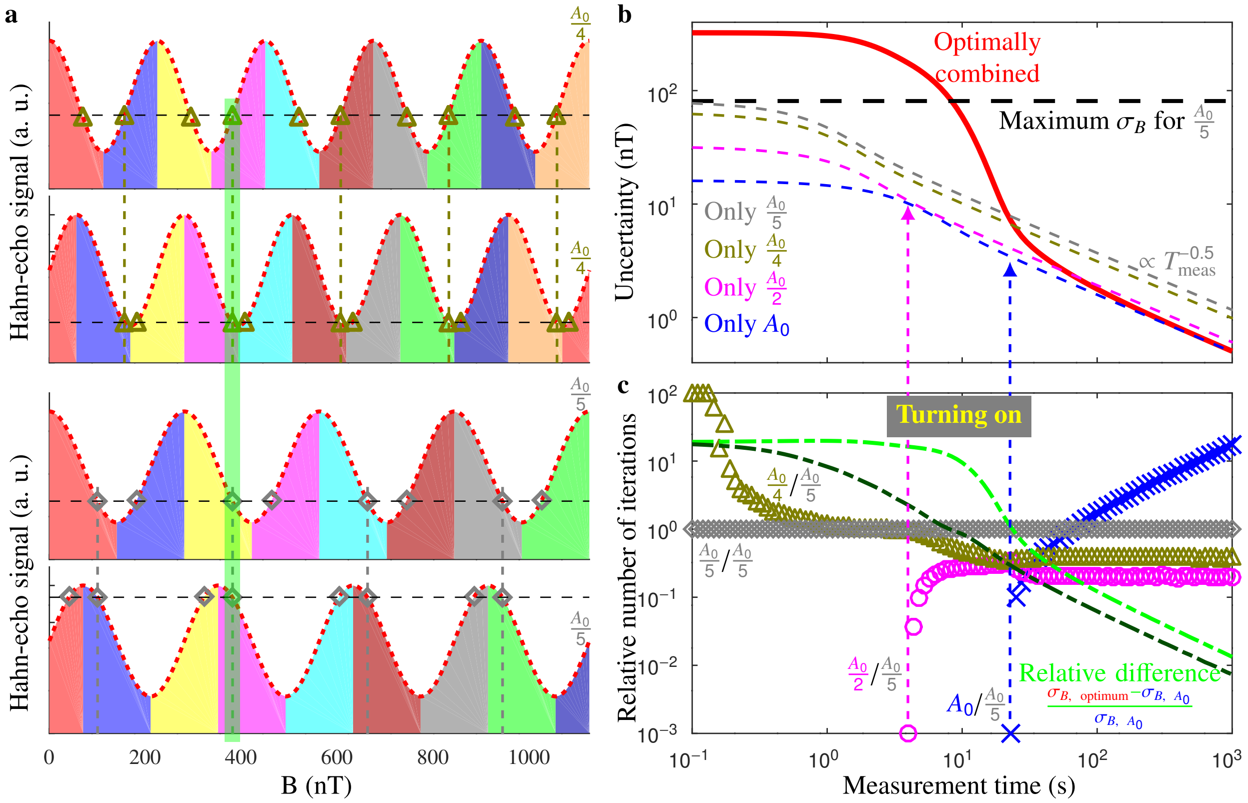

Supplementary Fig. 7. Range extension. **a** By combining areas that are not integer multiples of each other, the range goes beyond the range possible with just the smallest area (here $A_0/5$). The top two graphs show two phases for area $A_0/4$, and the bottom two graphs for smallest area $A_0/5$, with ranges indicated with different colours as in Supplementary Fig. 1a. The horizontal black dashed lines indicate measured signals for each sequence; the two phases of each area reduce the possible fields by two as indicated by the vertical dashed lines (and explained in Supplementary Note 2), while the combination of the two areas only leaves a single likely result (indicated by the green rectangle). Thus, their combined period and hence range, which follows from the inverse of the greatest common divisor of their frequencies, is here four times larger than the period of the smallest area. **b** Minimised uncertainty for a large-range sequence by optimally combining the subsequences (red line). The dashed lines give the uncertainty σ_B for the single-area sequences (A_0 blue, $A_0/2$ magenta, $A_0/4$ olive, $A_0/5$ grey). The maximum uncertainty for the smallest area $A_0/5$ (indicated with a horizontal black dashed line) is four times smaller than the maximum uncertainty of the combination, as expected for a fourfold increase of the range (see Supplementary Note 1). **c** The relative number of iterations for each area (A_0 blue crosses, $A_0/2$ magenta circles, $A_0/4$ olive triangles, $A_0/5$ grey diamonds kept at 10^0) for each measurement time T_{meas} to get the lowest uncertainty possible, which results in the red line in **b**. The upper light-green dashed line gives the relative difference between the most-sensitive small-range sequence (blue dashed line in **b**) compared to this optimally combined sequence. The ultimate uncertainty is still approached. The lower dark-green dashed line gives the relative difference between the most sensitive sequence compared to the optimally combined sequence of a set of areas with the same range and ultimate uncertainty but including the smallest area ($20A_0/20$, $8A_0/20$, $4A_0/20$, $2A_0/20$, $A_0/20$). They approach the ultimate uncertainty equally fast, but the latter is slightly ahead.

Supplementary Note 7: Algorithm implementation

In Supplementary Fig. 8, the absolute numbers of iterations for the result of Fig. 3b/c of the main text are displayed (as opposed to the relative numbers of iterations in main text's Fig. 3c). Since the absolute numbers of iterations are mostly increasing, if it is not necessary to fix the whole sequence, it is possible to measure optimally by choosing for which subsequence to increase the iterations during the measurement. This information depends on the system and the chosen areas only, and is thus known beforehand. Hence, the optimum uncertainty can be followed over measurement time.

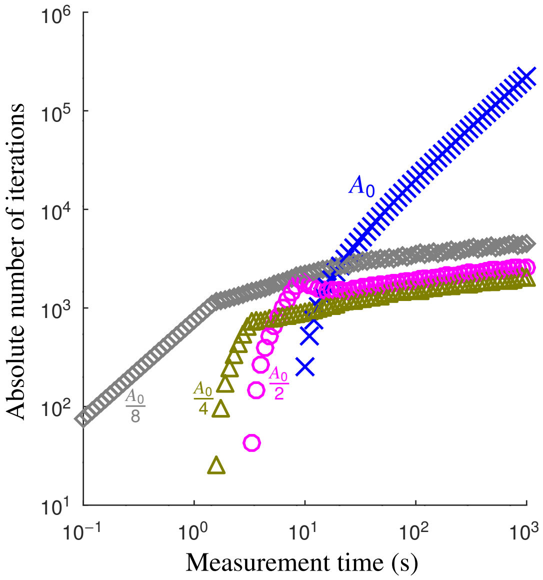

Supplementary Fig. 8. Algorithm implementation. The absolute number of iterations for each area (A_0 blue crosses, $A_0/2$ magenta circles, $A_0/4$ olive triangles, $A_0/8$ grey diamonds) for each measurement time to get the lowest uncertainty possible for the result of Fig. 3b/c in the main text. Since the absolute number of iterations are mostly increasing, it can be implemented by choosing for which area to increase the iterations depending on the current measurement time. The exception in monotonicity for $A_0/2$ is due to the non-optimum choice for the combination of areas: for example area $3A_0/4$, the area in between $A_0/2$ and A_0, is not part of the sequence (see Supplementary Note 4).

Supplementary Note 8: Small area measurements for AC

Given an AC field that affects the spin, a standard measurement looks like the first period in Supplementary Fig. 9. When reducing the area, the simplest option is to remain symmetric around the inflection point (so keeping the microwave π-pulse at the same place as shown in the second period of Supplementary Fig. 9), however, this is not a necessity. For example looking at an area of 2^8 times smaller than the initial one, we can do an equivalent measurement anywhere along the curve, since the DC part cancels. We just have to calculate the location of the microwave pulses to measure the required area, as illustrated in the third period in Supplementary Fig. 9. Around the linear parts, the pulse distance is rather similar to the one at the inflection point (which also means that no exact synchronisation is required in this region), while around the peak the delay is somewhat longer. Nonetheless, this is still less efficient compared to DC scaling (which area scales linearly with sequence length), but as advantage, with the same time delay between the π/2-pulses, a smaller area can be probed, hence a larger range is attained. Moreover, since for smaller areas the relative length of the overhead time increases, the advantage of the scaling of DC decreases, since this overhead will take most of the time.

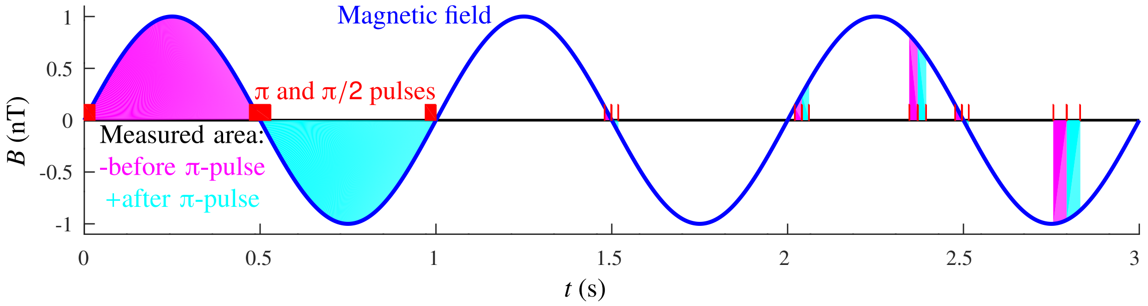

Supplementary Fig. 9. Measuring small areas. Example of an AC magnetic field B vs time t (blue line) with several areas measured (combination of magenta/cyan area-pairs, with magenta before the π-pulse, and cyan after) in between microwave pulses (red rectangles, not to scale). The first period of the AC field is measured fully in the standard way with microwave π/2-pulses at the beginning and the end of the period, and a π-pulse at the inflection point in between. The second period shows a measurement around the inflection point with an effective area which is 2^8 times smaller than the first area. The third period shows measurements of the same effective area but not specifically located at the inflection point, which works since the DC component cancels due to the refocussing π-pulse. The time delay between the π/2-pulses at the linear parts is almost the same as at the inflection point, while near the extrema a bit more time is required.

When combining multiple sequences with different areas and various phases, arranging these areas cleverly will efficiently use each period of the field. For example, the largest area can be measured from peak-to-peak (thus from $\pi/2$ to $5\pi/2$ for a sine), which has no additional time overhead, leaving the steep region for smaller areas, which have barely longer time delays in this region as discussed above. As example, we explore a basic sequence that would implement the largest-range measurement of main text Fig. 2c including all overhead times (so including laser pulses, waiting times, microwave pulses and idle times). Here, the chosen measurement time is beyond the steep region, and rounded relative numbers of iterations are chosen, resulting in $A_0 : A_n = 10 : 1$. Thus each area is measured once while the largest area is measured 10 times (compare with Supplementary Fig. 6). To keep it simple, the order of the areas is from the largest to the smallest, while the phase is changed after each smallest area. The complete design is plotted in Supplementary Fig. 11. This could be further optimised by changing the order of the areas.

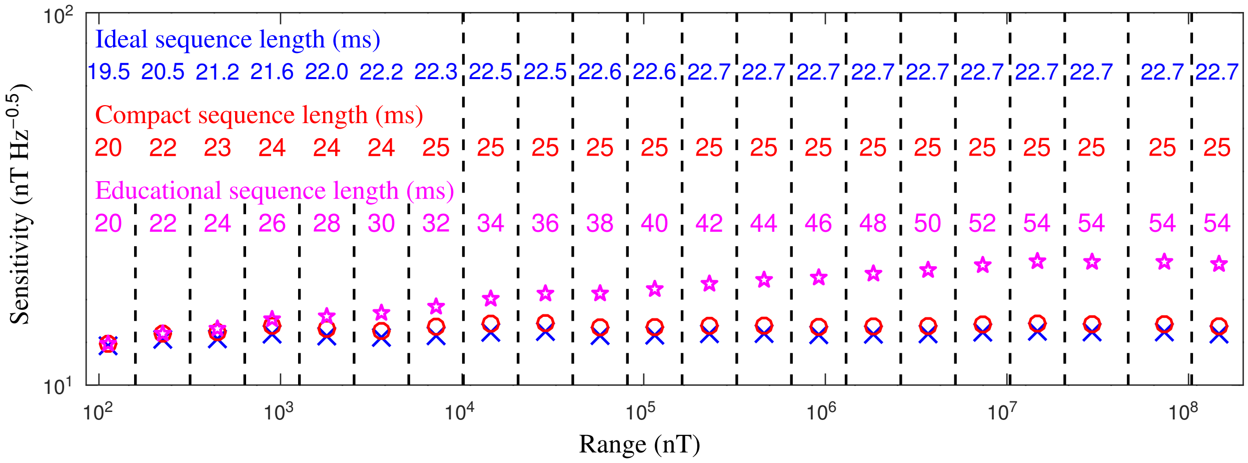

Supplementary Fig. 10. Overhead time effect. Sensitivity vs magnetic field range (at 2 kHz) for measurements comparing the effect of overhead time for different sequence choices. Please note that these measurements are performed on a different NV centre compared to main text Fig. 2c (but T_2 is also ~2 ms), and compact sequences are used for the measurements (see Supplementary Fig. 11). Magenta pentagons plot the averaged sensitivity of our algorithm at each range including all overhead time, while assuming a sequence would have been used where each area is measured during a new period of the field (the "educational sequence"). Red circles display the sensitivity including all overhead time for the basic sequence design used for these measurements (as in Supplementary Fig. 11, the "compact sequence"). Blue crosses give the sensitivity excluding all overhead time (the "ideal sequence"). For each sequence, the time length for each range is given above the respective data points. The difference between the latter two is rather small; please note that the sensitivity $\propto \sqrt{\text{measurement time}}$.

The effect of the overhead times (including idle times) is further explored with additional measurements which results are shown in Supplementary Fig. 10. Here, for each range sequences are designed similar to the one of Supplementary Fig. 11, while for the largest range the exact sequence from Supplementary Fig. 11 is applied. Also, the measurement time and resulting sensitivity per sequence are given for three scenarios. The first scenario is when assuming that each added area requires a separate period of the field, which means at each increase of the range (when another area is added), the measurement time increases by four periods (one for each phase). The second scenario is the actual measurement, thus the measurement time is the total data acquisition time and it follows from the compact sequences. The last scenario is the ideal scenario, for which all overhead time is completely ignored (so also idle time), while allowing to measure around the inflection points, thus further reducing the measurement time (as this gives the shortest time delay between the $\pi/2$-pulses). As follows from the results in Supplementary Fig. 10, even for this simple sequence design, the actual measurement time increases by about 10% compared to the ideal case, thus worsening the sensitivity (which is proportional to the square root of the measurement time) by merely 5%.

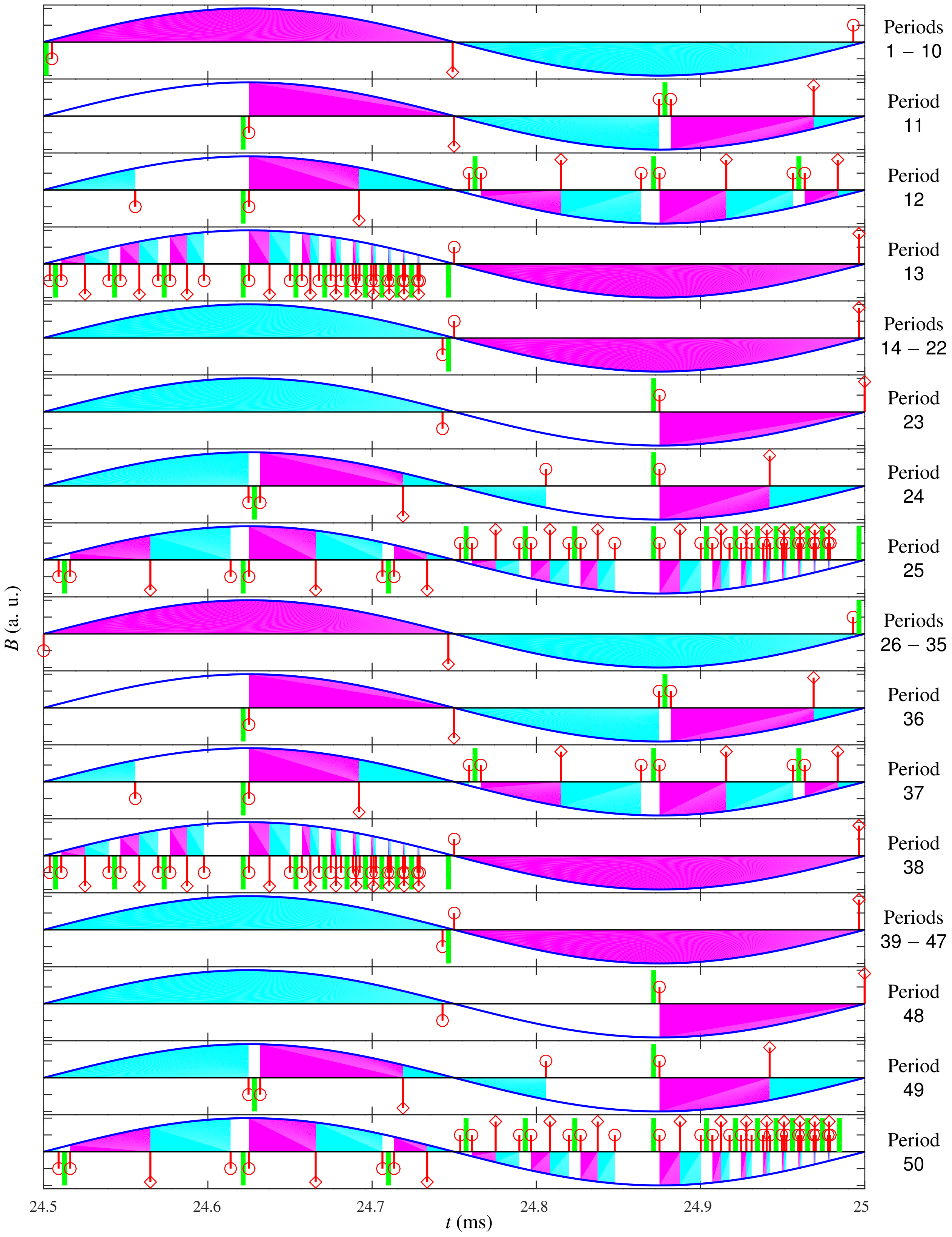

Supplementary Fig. 11. Compact sequences. Example compact sequence design for main text Fig. 2c for the largest range; the sequence is used for Supplementary Fig. 10. AC magnetic field B vs time t (blue line) with visualised measurement sequence including all overhead times: green rectangles for the laser pulses (to scale), red stems indicating the nanosecond-scale microwave pulses ($\pi/2$-pulses with halfway stems and circles at their top, π-pulse with full stem and diamond at its top). Furthermore, magenta areas are the parts between the first $\pi/2$-pulses and the π-pulses, and cyan areas the parts between the π-pulses and the second $\pi/2$-pulses. Please note that for clarity, during the first half of a period, the pulses are drawn below the x-axis, and during the second half above the x-axis. Even in this non-optimised sequence, while before some extrema measurement time is lost, most measurement time is utilised. For the smallest areas, the overhead time of the laser pulse and wait times becomes most significant.

Supplementary Note 9: Single result vs probability distribution

In Supplementary Fig. 12, simulated single results (which is the expected value of the field given its probability distribution) for multiple measurements with a long-measurement-time optimum point (thus most time is spent on the most sensitive subsequence) are displayed. Although few, there are results far away from the main peak, more likely located at the periods of the range of the most sensitive part of the sequence. However, if we compare the resulting probability distributions of results around the actual magnetic field with those of other results, we see that the former have a pronounced peak orders of magnitude away from the other peaks, while the latter have several strong peaks, thus they have larger individual standard deviations (thus uncertainties, see Supplementary Equation (2)). Hence, these results can easily be discarded by comparison with the expected uncertainty which can be derived from the sequence design.

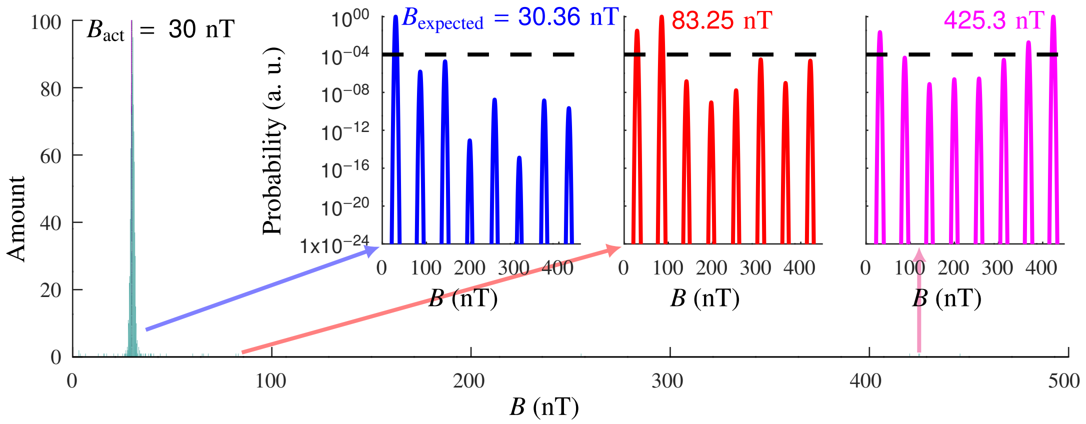

Supplementary Fig. 12. Probability distribution effect. Histogram of the expected values of the magnetic field B of 1,000 simulated measurements for the optimum sequence (the expected uncertainty derived from the sequence design is 0.80 nT) of a long measurement time with applied field B_{act} = 30 nT. The left inset shows a resulting distribution for a result around the peak (individual uncertainty is 0.96 nT), and the middle (individual uncertainty is 10 nT) and the right (individual uncertainty is 8.8 nT) insets for results far away. The black dashed lines in the insets are guides to the eye at a probability four orders of magnitude smaller than the peak probability. The displayed far-away results have the smallest individual uncertainties with respect to all far-away results, but nonetheless they are about 10 times worse compared to the expected uncertainty.

Supplementary References

1. Herbschleb, E. D. *et al.* Ultra-long coherence times amongst room-temperature solid-state spins. *Nat. Commun.* **10,** 3766 (2019).
2. Nusran, N. M. & Dutt, M. V. G. Optimizing phase-estimation algorithms for diamond spin magnetometry. *Phys. Rev. B* **90,** 024422 (2014).
